# Comparison of Methods for Competitive Tests of Pathway Analysis

**DOI:** 10.1371/journal.pone.0041018

**Published:** 2012-07-31

**Authors:** Marina Evangelou, Augusto Rendon, Willem H. Ouwehand, Lorenz Wernisch, Frank Dudbridge

**Affiliations:** 1 Medical Research Council Biostatistics Unit, Institute of Public Health, Cambridge, United Kingdom; 2 Department of Haematology, University of Cambridge, Cambridge, United Kingdom; 3 National Health Service Blood and Transplant, Cambridge, United Kingdom; 4 Wellcome Trust Sanger Institute, Cambridge, United Kingdom; 5 Faculty of Epidemiology and Population Health, London School of Hygiene and Tropical Medicine, London, United Kingdom; Aarhus University, Denmark

## Abstract

It has been suggested that pathway analysis can complement single-SNP analysis in exploring genomewide association data. Pathway analysis incorporates the available biological knowledge of genes and SNPs and is expected to improve the chances of revealing the underlying genetic architecture of complex traits. Methods for pathway analysis can be classified as competitive (enrichment) or self-contained (association) according to the hypothesis tested. Although association tests are statistically more powerful than enrichment tests they can be difficult to calibrate because biases in analysis accumulate across multiple SNPs or genes. Furthermore, enrichment tests can be more scientifically relevant than association tests, as they detect pathways with relatively more evidence for association than the remaining genes. Here we show how some well known association tests can be simply adapted to test for enrichment, and compare their performance to some established enrichment tests. We propose versions of the Adaptive Rank Truncated Product (ARTP), Tail Strength Measure and Fisher’s combination of p-values for testing the enrichment null hypothesis. We compare the behaviour of these proposed methods with the established Hypergeometric Test and Gene-Set Enrichment Analysis (GSEA). The results of the simulation study show that the modified version of the ARTP method has generally the best performance across the situations considered. The methods were also applied for finding enriched pathways for body mass index (BMI) and platelet function phenotypes. The pathway analysis of BMI identified the Vasoactive Intestinal Peptide pathway as significantly associated with BMI. This pathway has been previously reported as associated with BMI and the risk of obesity. The ARTP method was the method that identified the largest number of enriched pathways across all tested pathway databases and phenotypes. The simulation and data application results are in agreement with previous work on association tests and suggests that the ARTP should be preferred for both enrichment and association testing.

## Introduction

Pathways are groups of biologically related genes that act together in a specific biological process. Different pathways are responsible for different outcomes varying in complexity. An example of a biological pathway is the metabolic Gluconeogenesis pathway that is responsible for the generation of glucose. According to the KEGG database [1] this pathway consists of 65 genes, among them the genes GCK (glycokinase) and GPI (glucose-6-phosphate isomerase). GCK produces glucose-6-phosphate and GPI catalyses the reversible isomerization of glucose-6-phosphate. Both actions take part in energy pathways like Gluconeogenesis. Pathways may not work correctly due to a faulty signal received from one of the participating genes. Faulty pathways can result in disease; therefore pathway analysis is potentially important since it can reveal the underlying genetic structure of a disease. Pathway analysis incorporates the available biological knowledge of genes and simultaneously tests all pathway genes for association with a phenotype of interest. Several authors have discussed how pathway analysis can complement single-SNP analysis in exploring data from genomewide association scans (GWAS) [2], [3].

Pathway analysis is currently a popular topic and several methods have been published both for GWAS and a wider range of molecular analyses [3]–[11]. The proposed methods are distinguished by a number of aspects. Some of the methods require as their input data the raw genotype data, while other methods require only summary SNP or gene statistics. The methods also differ in their test statistics and on the way of assessing the significance of those statistics.

The methods can also be divided into association and competitive methods according to the null hypothesis tested [12]. The self-contained (or association) null hypothesis states that no pathway genes are associated with the phenotype. Testing the self-contained null hypothesis compares the statistics of the genes within the pathway to the null background. Therefore, association methods can be used in both pathway analysis and candidate gene analysis, since only the statistics from a selection of genes is required [2]. For example, some association tests combine the pathway gene p-values into a single p-value for the entire pathway, as done by Fisher’s product method, Truncated Product methods, and the Tail Strength Measure [11], [13], [14]. Regression models are also used to assess the joint significance of all pathway genes [15].

Alternatively, the competitive (or enrichment) null hypothesis is that the pathway genes are no more associated with the phenotype than the non-pathway genes. The competitive methods compare the statistics of the pathway genes with statistics of genes outside the pathway, to determine whether the pathway is more associated with the phenotype compared to other pathways. An enriched pathway can be defined as one whose genes are more strongly associated with the phenotype than those genes outside the pathway. Commonly used methods that identify enriched pathways are the Hypergeometric Test, Gene Set Enrichment Analysis and the SNP ratio test [3], [7], [9].

The two null hypotheses are related since by rejecting the competitive null hypothesis, the self-contained null hypothesis is also automatically rejected. In other words a pathway that is not associated cannot be enriched. The two hypotheses are equivalent only in the case that there are no associated genes outside the pathway. A method testing the self-contained null hypothesis will have more power than one testing the competitive null hypothesis, in that an association method will reject the null hypothesis for more pathways than a competitive method. The competitive null hypothesis in contrast makes a stronger statement, in this way sacrificing some of its power. The relationship and differences between the two null hypotheses have been discussed in detail by Goeman and Buhlmann [12].

Fridley et al [11] performed a simulation study examining the performance of existing and novel association methods for expression data. The methods considered can be divided into two categories: the methods that are based on summary gene statistics and those that perform a joint modeling of all the data for a given pathway. Among the methods considered, the Fisher product which combines the gene statistics into a single pathway p-value was shown to have the greatest power in detecting associated pathways.

In the context of GWAS, population stratification and/or cryptic relatedness may introduce some biases across the SNPs of the GWAS [16], [17]. These biases make the calibration of association methods in GWAS difficult. For example, population stratification inflates the SNP statistics by an average factor 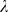. While this is usually ignorably close to 1 for single SNP tests, an appreciable bias may accumulate across multiple SNPs in a pathway. Although a Fisher product could be rescaled by an appropriate power of 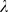, it is unclear in general how other association tests should be adjusted or calibrated. On the other hand, competitive methods detect pathway genes with relatively more evidence for association than the remaining non-pathway genes. Therefore, testing the competitive null hypothesis is more pragmatic in GWAS, and can be regarded as providing evidence for pathways of more biological relevance to the phenotype studied.

In recent years several methods have been proposed for pathway analysis that either test the self-contained null hypothesis or the competitive null hypothesis. A parallel development of the methods has been observed but there has been little overlap in the literature. In this paper we examine whether commonly used association methods can test the competitive null hypothesis by using an appropriate gene statistic. We propose using the scaled ranks of the gene p-values as the input data of the association methods, in order to adapt them to competitive tests. This approach can be used for any association method. Here we adapt Fisher’s Method (FM) [11], Tail Strength Measure (TSM) [14] and Adaptive Rank Truncated Product (ARTP) [6] to test the competitive null hypothesis. A simulation study was performed to compare the performance of the adapted association methods with commonly used competitive methods including the Hypergeometric Test [9] and Gene Set Enrichment Analysis (GSEA) [3]. This is the first time that these competitive tests have been compared to methods derived from association tests. In particular, the performance of the ARTP has not been compared to other pathway analysis methods except FM, and has not yet been widely applied to real studies. However, the results of our simulation study show that the adapted version of ARTP method is the most powerful in detecting enriched pathways.

In addition to the simulation study, the methods were applied to the data of two GWAS. The first study is a subset of the EPIC-Norfolk study [18] involving 3552 individuals for whom body mass index (BMI) was recorded. The second GWAS involves 500 healthy individuals and aims to find the genetic structure of platelet function which is described by four endpoints (phenotypes) [19], [20]. A detailed description of the two studies is given in the Methods section. The Reactome, KEGG and Biocarta pathways were downloaded for the analysis performed. Each pathway database was tested independently from the other databases for enrichment with the BMI phenotype of the first study and with the four phenotypes of the second study. The data application results concur with the simulation results in that the ARTP method is the most powerful in detecting enriched pathways. This is in agreement with the literature on association testing, and suggests that the ARTP method should be preferred for both association and enrichment testing.

## Methods

### Ethics Statement

Platelets GWAS: A cohort of 500 healthy subjects of predominantly Northern European origin was recruited from the National Health Service Blood and Transplant blood donor clinic in Cambridge after gaining informed, written consent in accordance with the Declaration of Helsinki (for details of the cohort see Jones et al (2007) [19]). The study was approved by the Huntingdon Research Ethics Committee.

**Table 1:** Mean type-I error of the methods.

Method	Mean Type-I Error
FM 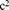	0.050
FM 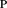	0.051
Hypergeometric 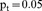	0.028
Hypergeometric 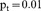	0.023
Hypergeometric 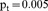	0.019
Hypergeometric 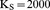	0.038
TSM 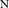	0.028
TSM 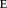	0.057
TSM 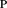	0.051
GSEA	0.049
ARTP	0.048
ARTP 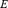	0.046

Mean type-I error of the methods across all null scenarios of the simulation study. TSM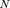 refers to the approximate Normal distribution of the TSM. FM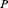 and TSM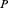 refer to the permutation procedures for estimating the significance of the FM and TSM statistic. ARTP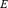 and TSM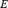 are the empirical distributions of ARTP and TSM respectively.

The pathway analysis performed was done anonymously for both EPIC-Norfolk and Platelets GWAS.

### Test Statistics

We propose to use scaled ranks of p-values in association methods, in order to test the competitive null hypothesis. The methods described here are Fisher’s method (FM), Hypergeometric Test, Tail Strength Measure (TSM), Gene Set Enrichment Analysis (GSEA) and Adaptive Rank Truncated Product (ARTP). This section ends with a description of the simulation study data and the data of the two GWAS used for testing the performance of the methods.

The association between a gene and the phenotype is often represented by the minimum p-value of the SNPs assigned to the gene, with appropriate adjustment for multiple testing in the gene. A number of other approaches are possible, but the minimum p-value has generally good properties and is most often used [21]. In our simulation we avoid this issue by assuming one SNP per gene, which will not alter our qualitative conclusions. The p-values of association between the GWAS genes and the phenotype are denoted by 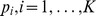 where 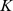 is the total number of genes in the study. The first step for computing the proposed gene statistic is to rank the gene p-values from the smallest to the largest. The statistic of the 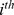 gene is denoted by 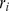 and equals the rank of the 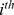 gene divided by K. Under the null hypothesis, the gene statistics 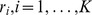 follow a discrete Uniform distribution with support 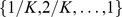.

**Table 2:** Mean power of the methods for the different pathway sizes.

Method	Pathway Size	Mean Power
ARTP 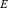	20	0.743
	60	0.892
	100	0.925
FM 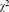	20	0.730
	60	0.889
	100	0.925
GSEA	20	0.639
	60	0.826
	100	0.867
TSM 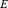	20	0.619
	60	0.837
	100	0.894
Hypergeometric 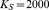	20	0.560
	60	0.729
	100	0.803

The mean power of the methods is computed for all the scenarios for the three different tested pathway sizes across all other variables.

**Figure 1:**
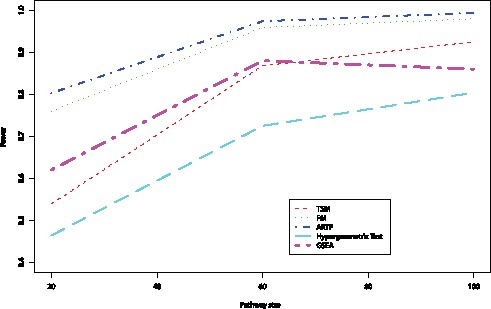
Power of the five methods for different pathway sizes. Plots illustrate the power of the methods when the total number of associated SNPs equals 100, the proportion of associated SNPs within each pathway is 0.4 and the effect sizes are 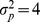 and 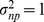.

#### Fisher’s method

Fisher’s method is a well established association method that combines the results from multiple statistical tests. The FM test statistic equals


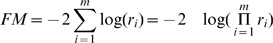
(1)

where 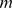 is the number of genes in the pathway. The FM test statistic follows a 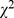 distribution with 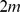 degrees of freedom when the gene statistics are independent and follow a continuous Uniform (0,1) distribution. The significance of the calculated FM test statistic can be estimated by either referring to the appropriate 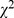 distribution or by comparing it to a set of null test statistics computed with randomized gene labels. The gene labels, which indicate whether a gene is or is not a member of the tested pathway, are randomly permuted and the FM test statistic is calculated based on the permuted gene labels. This procedure is repeated a large number of times to obtain the null permutation distribution. We used 1000 replicates. The p-value was then calculated as


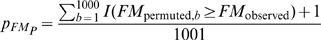
(2)

We were specifically interested in whether the 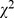 distribution was accurate when the gene statistics had a discrete distribution.

**Table 3:** Power of the methods as the proportion of pathway SNPs with effects changes.

Proportion ( 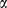 )	ARTP 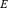	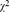 FM	GSEA	TSM 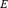	Hypergeometric 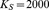
40%	0.940	0.909	0.881	0.691	0.659
60%	0.985	0.979	0.970	0.878	0.852
100%	1	1	0.990	0.996	0.985

Power of the methods for a pathway of size 20. 50 genes in total have effects. The effect size 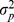 of the pathway genes is 4 and the effect size 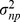 of the rest of the genes is 1.

**Figure 2:**
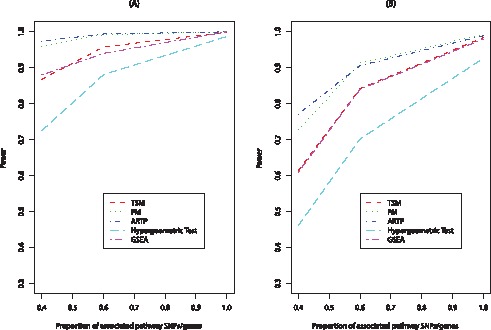
Power of the five methods for different proportions (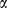) of associated SNPs within a pathway of size 60. Plots illustrate the power of the methods when the total number of associated SNPs equals 100 (plot (A)) and 200 (plot(B)), the effect sizes are 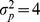 and 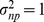 for both plots.

#### Hypergeometric test

We define a set of significant genes as those genes with p-values less than a threshold 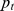. The Hypergeometric Test as a competitive method tests whether the pathway of interest contains more significant genes compared to those outside the pathway than expected by chance. Suppose that the pathway has 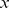 significant genes, then the p-value of enrichment of the pathway 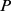 with 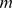 genes is given by


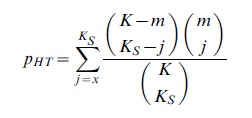
(3)

where 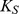 is the length of the significant genes list. The Hypergeometric Test assumes that the significant gene list is random and conditions on a fixed pathway. This is a one-sided test testing whether the pathway is enriched/over-represented within the list of most significantly associated genes with the phenotype. The Hypergeometric Test is a commonly used competitive test that is incorporated in a number of bioinformatics tools as discussed by Elbers et al [22].

#### Tail strength measure

The Tail Strength Measure proposed by Taylor and Tibshirani [14] is a measure of the statistical significance of the global null hypothesis of no gene effects. An advantage of the TSM is that it is asymptotically normally distributed. The TSM can be adapted to test the competitive null hypothesis by using the proposed gene statistic 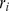. Firstly the 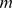 pathway gene statistics are ranked from the smallest to the largest such that 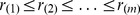. The TSM is then calculated as


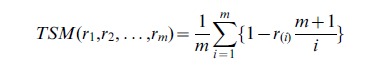
(4)

TSM calculates the deviation of each gene statistic from its expected value and large positive values of the TSM indicate evidence against the global null hypothesis, *i.e.* evidence that the pathway contains more significant genes than expected by chance. One of the advantages of the TSM is that, under the global null hypothesis, it is normally distributed for large enough 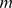, with mean zero and variance 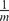. In practice pathway sizes are not large enough for validating the normal approximation. We computed an empirical distribution of TSM for pathways of size 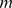 by randomly selecting 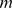 p-values from a Uniform 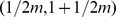 distribution, calculating the TSM statistic, and repeating the procedure 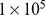 times. An alternative approach would be to use a discrete Uniform distribution in simulating the empirical distribution of the TSM. This would probably be more accurate than the continuous one but the latter works particularly well as we show in Results. In addition to the normal and empirical distributions of the TSM, we also compared the observed measure with a set of null measures computed with randomized gene labels, as described above for FM.

**Table 4:** Power of the methods for the different pathway sizes.

Pathwaysize	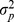	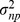	ARTP 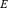	FM 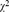	GSEA	TSM 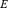	Hypergeometric 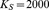
20	4	2	0.550	0.520	0.370	0.360	0.317
	4	1	0.803	0.760	0.620	0.539	0.464
	2	1	0.571	0.511	0.460	0.389	0.326
60	4	2	0.851	0.825	0.730	0.713	0.528
	4	1	0.974	0.959	0.880	0.868	0.725
	2	1	0.857	0.828	0.690	0.707	0.546
100	4	2	0.928	0.915	0.820	0.837	0.690
	4	1	0.994	0.980	0.860	0.925	0.804
	2	1	0.925	0.901	0.820	0.826	0.674

#### Gene set enrichment analysis

Wang et al [3] modified the widely-used method for microarray data, Gene Set Enrichment Analysis, to perform a gene-based pathway analysis of GWA data. GSEA, which is based on a weighted Kolmogorov-Smirnov-like running sum statistic, tests for over-representation of the pathway genes within the entire ranked list of genes. We use the negative logarithm of the gene p-values as the input gene statistics, denoted by 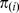. We have chosen this input gene statistic as we found it to give more numerically stable results than others, especially the gene p-value itself. The gene statistics are ranked from the largest to the smallest (with 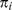 denoting the 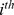 largest gene statistic). The weighted Kolmogorov-Smirnov-like running sum statistic is given by


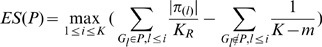
(5)

where 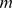 is the size of pathway 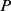 and 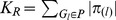. The significance of the statistic can be estimated by comparing it to a set of null statistics computed with randomized gene labels.

#### Adaptive rank truncated product

Yu et al [6] proposed the Adaptive Rank Truncated Product for performing a gene-based pathway analysis. Again we assume that statistics of the 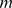 pathway genes are ranked such that 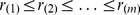. The original RTP statistic given by


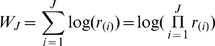
(6)

combines the 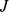 smallest gene statistics of the tested pathway. In the adaptive RTP the truncation point 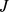 is chosen such that the p-value of 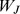 is minimised. The tested truncation points for a pathway of size 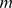 are all the integer values between 1 and 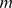. The RTP statistic simplifies to the FM test statistic when the truncation point 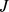 is fixed to 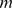. Simulated data with randomized gene labels are created for both calculating the significance of each 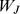 as well as estimating the appropriate truncation point 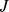. The RTP statistic


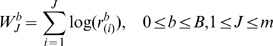
(7)

is calculated for each truncation point 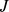, for both the observed data-set and each of the 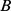 simulated datasets. Then Ge’s algorithm is used to estimate the p-value


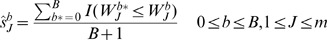
(8)

for each 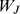 and for all data. The p-value for the ARTP statistic 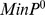 of the pathway is estimated as


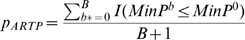
(9)

where


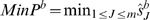
(10)

Ge’s algorithm [23] is used as suggested by Yu et al [6] for reducing the multiple-level permutation procedure into a single-level permutation procedure.

An alternative version for calculating the pathway p-value creates the simulated data-set using p-values from a continuous Uniform 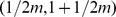 distribution instead of using permuted gene p-values. The ARTP significance based on this empirical distribution is denoted by ARTP*_E_*.

**Table 5:** Mean power of the methods that have a type-I error 5% across all simulated scenarios.

Method	Power
ARTP 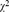	0.846
FM 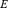	0.840
GSEA	0.768
TSM 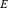	0.772
Hypergeometric 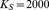	0.687

Mean power of the methods across all simulated scenarios.

**Figure 3:**
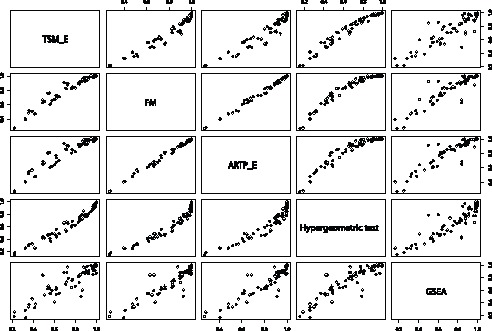
Pairwise scatterplot of power for the five methods across all simulated non-null scenarios.

### Simulation Study

A simulation study was performed to examine the performance of the adapted versions of the association methods FM, TSM and ARTP and of the competitive methods Hypergeometric Test and GSEA for testing the competitive null hypothesis. The type-I error and the power of methods were examined under various scenarios. To estimate the type-I error of the methods, data were created under the competitive null hypothesis that the pathway is not enriched. Then, data were created under the alternative hypothesis of enrichment to estimate the power of the methods. In the simulation study the effects of the following variables were examined: pathway size, total number of genes with effects, the variance of the effects of pathway genes, the variance of the effects of non-pathway genes and the proportion of pathway genes with effects.

**Table 6:** Pathway Analysis of BMI.

PathwayName	Size	ARTP 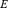	FM 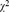	GSEA	TSM 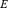	Hypergeometric 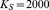
Biocarta:VIP Pathway	19	0.028	0.242	0.024	0.523	0.552

Table shows the nominal p-values of all five methods for the Biocarta VIP pathway. Biocarta VIP pathway has been reported as being significantly associated with BMI and the risk of obesity.

In the simulation study a genotype matrix 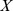 of size 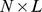 with entries 0, 1 and 2 was created. 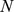 denotes the total number of individuals and 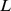 the total number of SNPs in the study. The minor allele frequency (MAF) of each SNP of the study was randomly selected from a Uniform (0, 0.5) distribution. The entries 0, 1 and 2 of each column of the genotype matrix 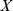 were sampled with probabilities equal to the genotype frequencies calculated from the MAF of each SNP under Hardy-Weinberg equilibrium.

In the subsequent steps of the simulation study, a single-SNP analysis is performed to test the association of each SNP with the response variable/phenotype. Each SNP is mapped to a unique gene so that the simulation regards SNPs and genes as equivalent.

**Table 7:** Performance of the methods when applied on the data of the GWAS.

Response	KEGG	Biocarta	Reactome
BMI	FM ( 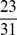 )	ARTP = GSEA ( 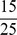 )	TSM ( 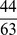 )
25 cm[1ex]Fibrinogen response to ADP	TSM ( 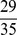 )	FM = GSEA ( 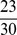 )	FM ( 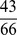 )
Fibrinogen response to collagen	FM = TSM ( 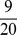 )	ARTP ( 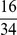 )	ARTP ( 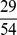 )
P-selectin response to collagen	GSEA ( 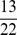 )	ARTP = FM = TSM ( 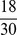 )	ARTP ( 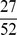 )
P-selectin response to ADP	GSEA ( 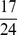 )	GSEA ( 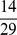 )	ARTP ( 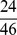 )

Table shows the method that identifies the largest number of pathways with nominal p-value less than 0.05 for each phenotype and database. The numbers in the brackets represent the number of enriched pathways identified by the equivalent method divided by the total number of enriched pathways identified by all the tested methods.

For testing the type-I error of the methods, a number of SNPs were randomly selected from the 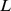 SNPs of the study. Following a quantitative genetic model, these SNPs were each assigned a random effect, denoted by 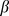, drawn from a Normal distribution with mean zero and variance 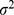. The variance values 1, 2 and 4 were considered. Other SNPs had no effect. The pathway SNPs were also randomly selected. Selection of the pathway members and the SNPs with effects were independent steps that could be done in any order.

In the non-null scenarios the first step was to select the pathway SNPs. A proportion (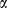) of pathway SNPs/genes were selected as having non-zero effect on the response variable. We assume the effect of pathway genes is stronger than of non-pathway genes by drawing pathway effects from N(0,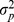) and non-pathway effects from N(0,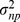) with 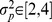, 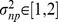 and 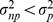. The greater variance of the effects within the pathway represents an enrichment of association compared to genes outside the pathway, as in these scenarios the effect sizes of the genes within the pathway are larger than the non-pathway genes.

For both cases, the last step in constructing the data of the study is to calculate the response variable 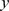 for the 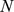 individuals. An additive SNP model is assumed for each individual, such that the response of individual 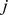 is calculated as:


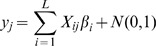
(11)

where effectively only the SNPs with effects play a role in the value of 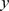.

For all null and non-null scenarios of the simulation study, pathway sizes of 20, 60 and 100 SNPs were tested, which is typical of current databases. The total number of SNPs with effects varied with 50, 100 and 200 SNPs. The total number of individuals tested was 100 and the total number of SNPs/genes was 20000. These numbers are smaller than found in a typical GWAS but were chosen to achieve power levels in the relevant range of 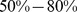, while allowing the simulation to complete in a reasonable time. One thousand simulated datasets were created for each scenario tested, giving standard errors for the estimated power of 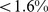. All tested scenarios can be found in table S1.

### Data Application

Further to the simulation study data, the methods were applied on the data of two GWAS for finding their enriched pathways. The phenotype of the first study is BMI which was measured for 3552 individuals living in Norfolk,UK. This GWAS is a subset of the EPIC-Norfolk study [18], which is part of the European Prospective Investigation into Cancer and Nutrition (EPIC) (http://epic.iarc.fr/) study and involves over 30,000 individuals living in Norfolk, UK.

The second GWAS aims to find the genetic architecture of platelet function. Platelets play a key role in thrombus formation during normal hemostatic responses to injury and atherothrombotic events. Platelet function as discussed by Jones et al [19], [20] can be measured by the four endpoints (phenotypes): p-selectin and fibrinogen responses to both ADP and collagen agonists. These four phenotypes were measured for the 500 individuals previously described by Jones et al [19], [20]. The 500 individuals of the study were genotyped using the Illumina610 chip. Standard quality control filters were applied to both SNPs and individuals, with 480 individuals and 544,078 SNPs retained for analysis.

The following steps were taken before applying the methods to the data of the two studies. The SNPs of each study were mapped to genes according to physical distance: a SNP was mapped to the closest gene whose coding sequence began 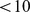
kb from the SNP. The minimum p-value of all the SNPs mapped to a gene was then assigned to the gene. As discussed by others [3], larger genes are more likely to be assigned a smaller p-value. To avoid any biases because of the gene size, the gene p-values were adjusted using phenotype label permutations, as follows.

The phenotype labels were permuted and single-SNP analysis was re-performed. The minimum p-value of all the SNPs mapped to a gene was assigned to the gene. The adjusted minimum p-value of the gene is then calculated as


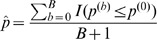
(12)

where 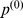 denotes the observed gene p-value and 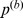 denotes the gene p-value at the 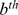 permutation. One thousand permutations were performed.

Gene sets were downloaded from the Molecular Signatures Database of Broad Institute (http://www.broadinstitute.org/gsea/msigdb/index.jsp). The gene-sets of the pathway databases Reactome [24], [25], KEGG [1] and BioCarta (http://www.biocarta.com/) were tested for enrichment. Each database was tested independently from the other databases for enrichment with each one of the five phenotypes of the two GWAS.

## Results

### Simulation Study

We performed simulations consisting of several null and alternative scenarios according to the hypothesis under which the response variable was computed. In the null scenarios the computed response was calculated using a number of genes with non-zero effects. The pathway genes were randomly selected from the full list of genes. The pathway sizes and the number of genes with non-zero effects as well as the effect size of the genes were varied. In the alternative scenarios the pathway genes selected were divided into pathway genes with effects and pathway genes with no effects. The response was computed including the effects of the pathway genes and a number of non-pathway genes that were randomly selected from the remaining list of genes. The association of the pathway genes with the response was assumed to be greater than the association of the rest of the genes. Different proportions of pathway genes with non-zero and zero effects were examined as well as different sizes of effects.

In the null scenarios the pathway size, the total number of genes with effects and the variance of the effect size 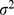 were varied (see Methods for full details). Table 1 shows the mean type-I error of the methods among all tested scenarios. The ARTP (using both permutations and its empirical distribution), GSEA and FM (using both permutation and asymptotic distribution) have an appropriate 5% error. The low type-I error of the TSM shows that the normal approximation is invalid for the small pathways examined in the simulation study. On the other hand, the empirical and permutation distributions of the TSM have a 5% type-I error. The Hypergeometric Test also has a low type-I error for the three different p-value thresholds examined, owing to the fact that it follows a discrete null distribution and an exact 5% rejection rate may not always exist. An appropriate type-I error of the Hypergeometric Test was observed when the number of significant genes was increased to 2000 for all tested scenarios, which we denote by Hypergeometric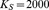. Based on these observations we referred only to the asymptotic distribution of the FM test statistic (FM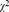) and empirical distributions of TSM (TSM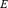) and ARTP (ARTP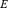) in the alternative scenarios of the simulation study.

The power of FM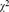, Hypergeometric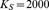, TSM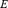, GSEA and ARTP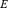 was examined in non-null scenarios. In the non-null scenarios, the pathway size, the total number of genes with effects, the proportion of pathway genes with effects and the size of pathway and non-pathway effects were varied. Table 2 shows the mean power of the methods for three different pathway sizes averaged over the proportion of significant genes in the pathway and the effect sizes in and out of the pathway. The power of all methods increases as the pathway size increases, reflecting both the increased proportion of pathway genes among the fixed total of associated genes, and the decreased proportion of non-pathway genes. In addition, figure 1 shows the power of the methods when the total number of associated SNPs equals 100, the proportion of associated SNPs within each pathway is 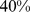 and the effect sizes are 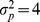 and 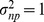. ARTP has the greatest power for all pathway sizes. FM has a lower power than ARTP for smaller pathways but as the pathway size increases, the power of FM increases reaching the power of ARTP. GSEA outperforms TSM for smaller pathways but as the pathway size increases TSM has a greater power than GSEA. Hypergeometric Test has the lowest power among all tested pathway sizes.

The power of all the methods increases as the proportion of pathway genes with effects increases. While keeping the total number of genes with effects fixed and increasing the proportion of pathway genes with effects, the number of non-pathway genes with effects decreases and as a result the power of the methods increases (Table 3). Table 3 shows how the power of the methods changes as the proportion (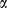) of pathway genes with effects increases. The ARTP has a significantly higher power than the other methods for small proportions of associated genes within a pathway. FM has a comparable power with ARTP especially in cases where the proportion of pathway genes with effects is high as for example in the case of all the pathway genes having an effect. TSM outperforms GSEA when all the pathway genes have an effect on the response while GSEA outperforms TSM when a smaller proportion of the pathway genes have an effect on the response. The Hypergeometric Test has the smallest power for all tested proportions. Figure 2 shows how the power of the methods changes with 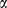 for a pathway of 60 genes while the effect sizes are 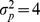 and 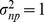. The first plot corresponds to a total number of 100 genes with effects (in and out of the pathway) and the second plot to a total number of 200 genes with effects. As it can be seen, as 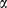 increases the power of the methods increases, and the power of the five methods is greater in the first plot compared to the second plot indicating that the methods are more powerful when the enriched pathways include most of the genes with effects of the study.

Table 4 shows the dependence of the power on the effect size variance 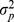 of the pathway genes and effect size variance 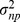 of the non-pathway genes. As can be seen from table 4 both the value of 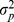 and the ratio between 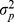 and 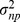 have an effect on the power of the methods. The methods attain the highest power when 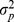 equals 4 and 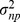 equals 1. The ARTP has the highest power followed by FM and GSEA. Table 4 also shows that the power of the methods increases as the pathway size increases.

Table 5 shows the mean power of the methods: FM, Hypergeometric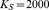, TSM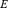, GSEA and ARTP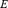 across all simulated scenarios that can be found in the Table S1. As can be seen from table S1, ARTP has the highest power in most of the cases and it has the highest mean power across all simulated scenarios (Table 5). FM is the second most powerful method with some cases having equal or greater power than ARTP. GSEA and TSM methods follow. The method with the lowest power in all tested scenarios is the Hypergeometric Test. Figure 3 displays a pairwise scatterplot of the five methods across all non-null simulated scenarios. The points of the scatterplots of ARTP and FM against the rest of the methods are above the diagonal line indicating that the ARTP and FM have higher power than the rest of the methods. The points in the scatterplot of GSEA against TSM (or vice-versa) fall very close to the diagonal indicating that the power of the two methods is very similar across all non-null simulated scenarios (figure 3). Similarly, the power of ARTP is very close to the power of FM but it is slightly higher.

### Data Application

In addition to the simulation study the five methods: FM, Hypergeometric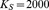, TSM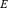, GSEA and ARTP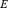 were applied to real data for finding enriched pathways for the phenotypes of two GWAS. The two studies aim to find the genetic structure of BMI and platelet function, respectively. Platelet function as discussed by Jones et al [19], [20] is described by four phenotypes: p-selectin and fibrinogen responses to both collagen and ADP agonists. A gene-based pathway analysis was performed on the five phenotypes: BMI, p-selectin response to ADP, p-selectin response to collagen, fibrinogen response to ADP and fibrinogen response to collagen (see Methods for full details). The pathways of Reactome, KEGG and Biocarta databases were downloaded. Each database was tested independently for enrichment with each one of the five phenotypes. Tables S2 and S3 show the pathways identified as enriched by the five methods for both BMI and platelets GWA data. The pathways given in the tables have been identified by at least one of the methods as enriched (*i.e.* with a nominal p-value less than 0.05).

The pathway analysis of the BMI phenotype replicated the main result of the pathway analysis performed by Liu et al [26], in which the Vasoactive Intestinal Peptide (VIP) pathway was identified as significantly associated with BMI and the risk of obesity. The ARTP and GSEA identified the VIP Biocarta pathway as being significantly associated with BMI whereas the other methods did not (table 6 and table S2).

Table 7 shows the method that identified the largest number of enriched pathways for each database and phenotype. The fractions in the brackets represent the number of enriched pathways identified by the corresponding method divided by the total number of enriched pathways identified by the five methods. ARTP is the method that appears in most of the table cells. ARTP is the method that identified the largest number of associated pathways with a nominal p-value less than 0.05 in most of the cases. ARTP is followed by FM and GSEA. TSM appears less often in the table and the Hypergeometric Test does not appear at all. No pathways with a p-value less than the corresponding Bonferroni correction p-value have been identified by the methods ARTP, FM, GSEA and TSM. On average ARTP followed by FM performed better than the other methods. This conclusion is derived from looking at the total number of p-values less than 0.05 which suggests that there are enriched pathways amongst them. ARTP method has the greatest overall power than the other methods, even if it has not identified any individual pathways contributing to the gain of this power.

## Discussion

We performed a simulation study to compare the performance of methods that test the competitive null hypothesis. Two commonly used competitive methods, Hypergeometric Test and GSEA, and adapted versions of the association methods FM, TSM and ARTP were examined. This is the first time that these association methods have been considered for competitive testing, allowing a wider comparison of competitive methods than has previously been possible.

We propose using the scaled ranks of the gene p-values as gene statistics used by association methods for testing the competitive null hypothesis. This novel feature enables the use of analytic and/or empirical distributions of the association test statistics, and the simulation study showed that these distributions have the correct type-I error rate. The proposed gene statistics follow a discrete Uniform distribution between 1 over the total number of genes in the study and 1, and deviation from uniformity implies enrichment of the pathway. The use of the scaled ranks as the input gene statistics provides a direct comparison between the pathway and non-pathway genes. While an indirect comparison could be performed by simply evaluating the association statistics under gene-label permutations, our approach allows rapid assessment of significance using analytic or empirical distributions. The scaled ranks do not depend on the underlying distribution of the gene p-values, and hence on any bias contained therein, and do not depend on how the gene-level association is derived from single-SNP tests. Extensive literature exists on methods for summarizing gene-level association. The power of these methods depends on the LD between the SNPs in the gene as well as on their allele frequencies and effect sizes [21], [27]. We avoided this issue by assuming one SNP per gene, but our results will hold qualitatively for any well calibrated gene-level test. In other words, as the methods tested here use gene statistics as their input statistics, as long as the gene statistics have been adjusted to take both LD and gene size into consideration, those factors should not play a role in the results of the pathway analysis. If there are systematic differences in LD and/or effect size between pathway and non-pathway genes, then more powerful methods could be developed to exploit such differences, and this is a promising direction for future work.

It was observed that the Hypergeometric Test can have a lower rejection rate than the nominal significance rate. This conservative property results from the discrete null distribution of the test, and is more profound when the sample size is small [28], in our case with small pathways. The appropriate type-I error of 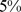 was found by fixing the number of significant genes to 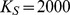 for all tested null scenarios. Other approaches such as mid p-value introduced by Lancaster [29] are available to ensure a correct type-I error of the Hypergeometric Test. Despite this aspect, which can reduce power, and the need to pre-specify a significance threshold (or to consider multiple thresholds with an appropriate penalty), the Hypergeometric Test is very commonly used as an enrichment test. In our simulation study, the non-null scenarios tested involve simulated pathways with genes with relatively larger effects than the ones outside the pathways. Non-null scenarios in which enriched pathways have an overabundance of significant genes with the same effects as the non-pathway genes were not explicitly tested here. However we expect the same general conclusions to apply since the net result is again an increase in total variance of effect sizes within the pathway.

The results of the simulation study suggest that the ARTP can and should be used as a more powerful test of enrichment. Our simulation results also agree with the findings of Tintle et al [9] who showed that the Hypergeometric Test is less powerful than the GSEA. In addition they showed that the GSEA is less powerful than the SUMSTAT method which is conceptually equivalent to the FM tested here. Furthermore, our results agree with the findings of Fridley et al [11] who showed that FM is the most powerful method compared to TSM and other methods for testing the association null hypothesis. However neither group of authors considered the ARTP, which we have shown to be more powerful than FM when adapted to enrichment testing. This result concurs with those of Yu et al [6] who compared ARTP to FM but not to other methods such as TSM and GSEA.

In addition to the simulation study, the methods were applied to the data of two GWAS. The pathway analysis of BMI replicated the main result of the pathway analysis performed by Liu et al [26] that identified the VIP pathway as significantly associated with BMI. In our data however, the result was only replicated using the ARTP and GSEA methods. In addition, a gene-based pathway analysis was performed on the GWAS that aims to find the genetic structure of platelet function. The results by applying the methods on the four endpoints (phenotypes) that describe platelet function concur with the simulation results that ARTP is the more powerful method. The ARTP was the method that identified the largest number of enriched pathways for most of the tested phenotypes and pathway databases.

The adapted version of ARTP was shown to be the most powerful for detecting enriched pathways. The ARTP is an extension of the RTP statistic, which considers the 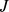 best gene statistics of every tested pathway. The use of Ge’s algorithm with ARTP has the advantage that a single level of permutation is needed for estimating the best 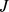 for each pathway and the p-value of enrichment between the pathway and the phenotype. We were surprised that the TSM and GSEA did not have comparable power to the ARTP. Conceptually the tests are similar, looking for a deviation from uniformity in the p-value distribution, in scenarios in which the deviation tends to lie in the tail. The TSM has the advantage of a known asymptotic distribution. However, despite its theoretical appeal it appears to have inferior power to alternative methods considered by us and other authors.

The FM statistic equals the RTP statistic when 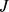 is the total number of genes, as discussed by Dudbridge and Koeleman [13]. It has been shown that ARTP has better power than FM in realistic scenarios of association testing. Combining all the above, we recommend the ARTP as the most powerful method for testing both association and enrichment null hypotheses.

## Supporting Information

Table S1 **Type-I error rates and power of the methods for the various scenarios tested in the simulation study.**
(XLS)

Table S2 **Pathways identified as enriched by the five methods for BMI phenotype.**
(XLS)

Table S3 **Pathways identified as enriched by the five methods for the four endpoints of platelet function.**
(XLS)
